# Anti-stroke biologics: from recombinant proteins to stem cells and organoids

**DOI:** 10.1136/svn-2023-002883

**Published:** 2024-01-29

**Authors:** Zhu-Wei Miao, Zhi Wang, Si-Li Zheng, Shu-Na Wang, Chao-Yu Miao

**Affiliations:** 1 Department of Pharmacology, Second Military Medical University/ Naval Medical University, Shanghai, China

**Keywords:** stroke, brain, thrombolysis

## Abstract

The use of biologics in various diseases has dramatically increased in recent years. Stroke, a cerebrovascular disease, is the second most common cause of death, and the leading cause of disability with high morbidity worldwide. For biologics applied in the treatment of acute ischaemic stroke, alteplase is the only thrombolytic agent. Meanwhile, current clinical trials show that two recombinant proteins, tenecteplase and non-immunogenic staphylokinase, are most promising as new thrombolytic agents for acute ischaemic stroke therapy. In addition, stem cell-based therapy, which uses stem cells or organoids for stroke treatment, has shown promising results in preclinical and early clinical studies. These strategies for acute ischaemic stroke mainly rely on the unique properties of undifferentiated cells to facilitate tissue repair and regeneration. However, there is a still considerable journey ahead before these approaches become routine clinical use. This includes optimising cell delivery methods, determining the ideal cell type and dosage, and addressing long-term safety concerns. This review introduces the current or promising recombinant proteins for thrombolysis therapy in ischaemic stroke and highlights the promise and challenges of stem cells and cerebral organoids in stroke therapy.

## Introduction

 Stroke, a cerebrovascular disease, is the second most common cause of death and the leading cause of disability with high morbidity worldwide.^1^,^3^ There are two types of strokes, ischaemic and haemorrhagic stroke, and approximately 70% of patients who had a stroke are ischaemic stroke. For the treatment of acute ischaemic stroke, alteplase is currently the sole thrombolytic agent approved by all regulatory agencies. Because of the narrow therapeutic time window, contraindications and complications, merely a small fraction of patients who had a stroke receive alteplase treatment.^1 4^ Moreover, due to the low rate of recanalisation, the effectiveness of alteplase therapy is limited.^2 4^ Recently, in cases of patients who had an acute ischaemic stroke with large artery occlusion, the combination of intravenous alteplase thrombolysis and endovascular thrombectomy has increased the rate of recanalisation, surpassing intravenous alteplase thrombolysis alone.^2 4^ However, many patients with this combination therapy will still be functionally dependent or disabled, due to delayed complete recanalisation resulting in brain tissue injury, or just partial recanalisation of visualised arteries, or the presence of microthrombi at the capillary level, causing incomplete tissue reperfusion. Also, certain regions lack access to or encounter difficulty in obtaining endovascular thrombectomy. Thus, there is an urgent requirement for developing new drugs to stroke therapy.

Biologics (biological drugs or biopharmaceuticals) are biotechnology-based medical products that are produced from living organisms/cells. They mainly contain recombinant proteins, vaccines, antibodies, gene therapy and cell therapy products. In current treatment for ischaemic stroke, alteplase as the sole drug has already been showed its distinctive advantage. However, requirements for improved efficacy and safety of thrombolysis have been highlighted for more than 25 years, leading to further study into systemic reperfusion for ischaemic stroke therapy. On the other hand, the strategies of regenerative medicine using cell or tissue therapy have been tested for repair of stroke brain injury. Hence, this review aims to discuss biologics for stroke therapy, including recombinant proteins for thrombolysis, stem cells and organoids for repairment and regeneration.

## Recombinant proteins for stroke therapy

**Figure 1:**
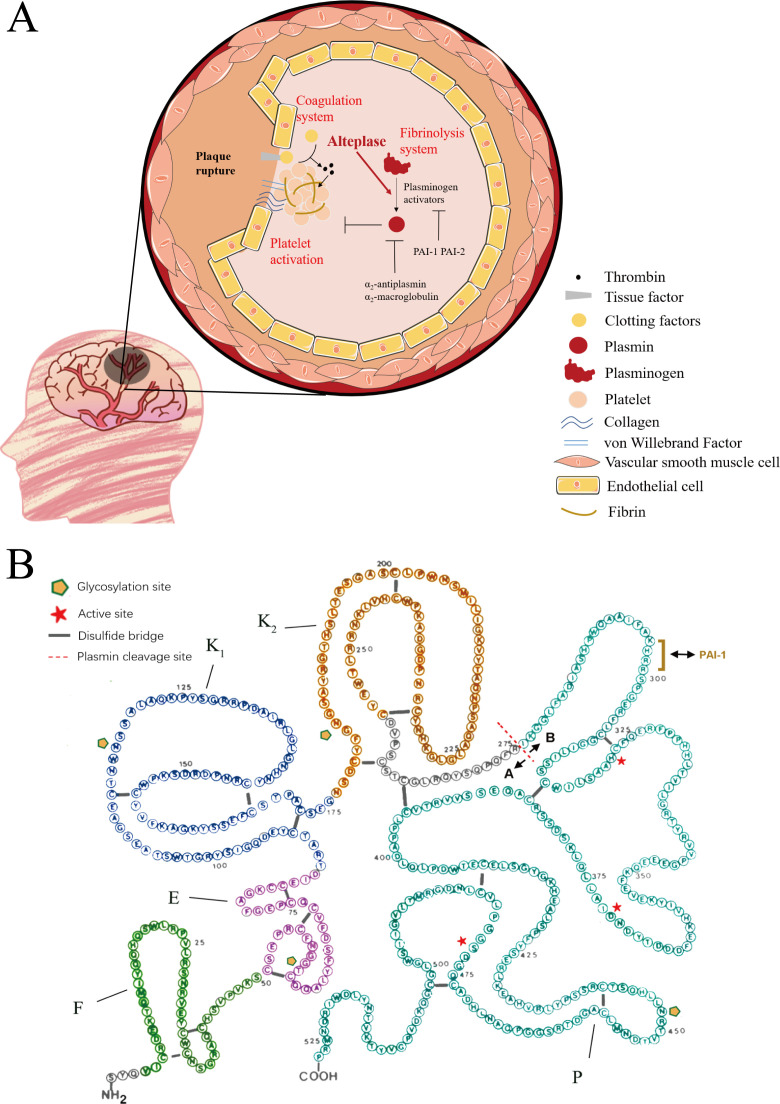
Thrombosis and fibrinolysis in acute ischaemic stroke (**A**) and primary structure of human tissue plasminogen activator (alteplase) (**B**). PAI, plasminogen activator inhibitor.

Arterial thrombosis is the main cause of ischaemic stroke. Three major systems play important roles in this process by which arterial thrombi form in atherosclerotic plaque rupture (figure 1).^5^ First is platelet activation. Following vascular injury, the subendothelial extracellular matrix (collagen and von Willebrand factor) will be exposed, which triggers the activation and aggregation of a platelet monolayer via their receptor glycoproteins (GP) GPVI and GPIb/V/IX. Second, coagulation plays a crucial role in this process. Deeper damage of endothelial cells leads to release of tissue factor (TF), TF combined with VII clotting factor causes the conversion of prothrombin to thrombin, in turn thrombin promotes fibrin generation, platelet activation and clot formation. Third, fibrinolysis system cleaves fibrin fibres formed in thrombus, avoiding thrombus further expanding. During the fibrinolysis, plasminogen is activated into plasmin by several endogenous activators, such as tissue-type plasminogen activator (tPA), urokinase-type plasminogen activator (uPA), kallikrein and neutrophil elastase, and then plasmin cleaves fibrin. In this process of fibrinolysis, plasmin can be inhibited by α_2_-antiplasmin and tPA can be inhibited by plasminogen activator inhibitor-1 (PAI-1) and PAI-2.

**Figure 2:**
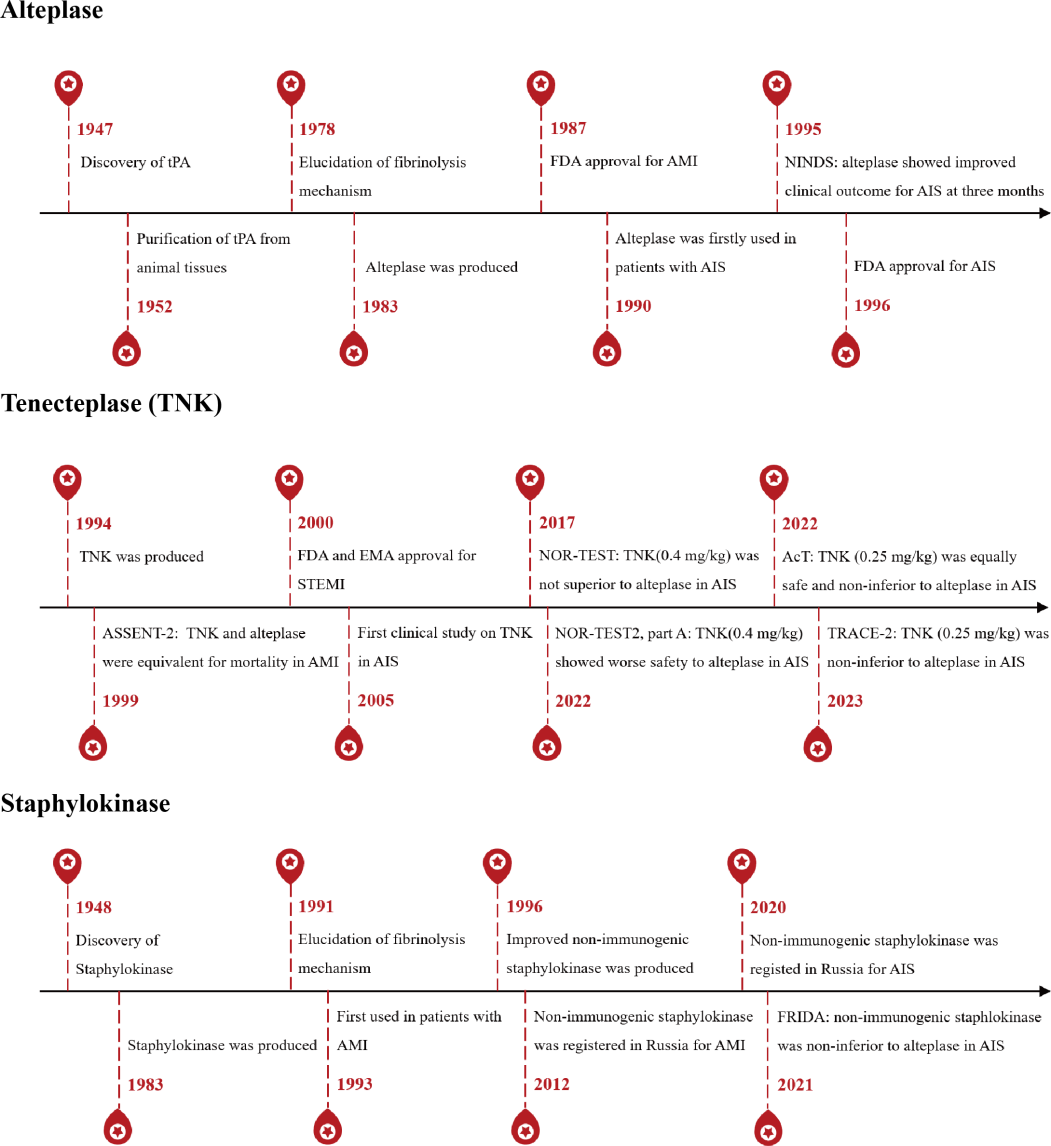
Development of three thrombolytic agents. AcT, alteplase compared with tenecteplase; AIS, acute ischaemic stroke; AMI, acute myocardial infarction; ASSENT-2: Assessment of the Safety and Efficacy of a New Thrombolytic Regimens 2; EMA, European Medical Agency; FDA, Food and Drug Administration; FRIDA: Non-immunogenic recombinant staphylokinase versus alteplase for patients with acute ischaemic stroke 4.5 hours after symptom onset in Russia; NINDS, National Institutes of Neurological Disorders and Stroke trial; NOR-TEST, The Norwegian Tenecteplase Stroke Trial; STEMI, ST-elevation myocardial infarction; TNK, tenecteplase; tPA, tissue-type plasminogen activator; TRACE-2, Tenecteplase Reperfusion therapy in Acute ischaemic Cerebrovascular Events-2.

For clinical treatment of acute ischaemic stroke, alteplase, a recombinant protein of human tPA, is currently the only thrombolytic agent. Other thrombolytic agents such as tenecteplase, a recombinant protein of alteplase variant and non-immunogenic staphylokinase, a recombinant protein of staphylokinase variant, are also promising in clinical trials. Here, we introduce these three thrombolytic agents about their brief history, protein characteristics and function mechanism (figure 2 and table 1).

**Table 1:** Comparison of key features among three thrombolytic agents for clinical use or trial in acute ischaemic stroke

	Alteplase	Tenecteplase	Non-immunogenic staphylokinase
Structure	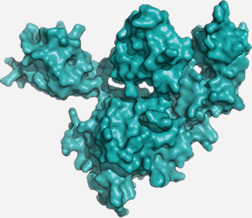	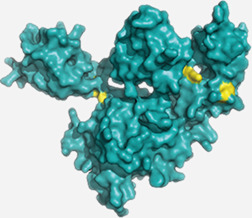 Thr103→Asn,Asn117→Gln,Lys296-His-Arg-Arg→Ala-Ala-Ala-Ala	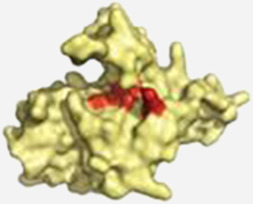 Lys74→Ala,Glu75→Ala,Arg77→Ala
Molecular weight	70 kDa	70 kDa	16.5 kDa
Dose and administration	0.9 mg/kg (≤90 mg)10% bolus and 90% infusion over 60 min	0.25 mg/kg (≤ 25 mg)Single bolus	10 mgSingle bolus
Fibrin-selectivity	Low	High	High
Inhibition by PAI	Yes	No	No
Plasminogen activation	Direct	Direct	Indirect
Half-life	5 min	22 min	6 min
Immunogenicity	No	No	No
Cost	High	High	Low
Treating acute ischaemic stroke	Clinical use	Clinical trial	Clinical trial

PAI, plasminogen activator inhibitor

### Natural tPA and its recombinant protein alteplase

The first observation of tPA was demonstrated in 1947, when it was observed that animal tissues contain an agent capable of activating plasminogen, initially referred to as fibrinokinase. Later, the substance was purified from various tissues and was confirmed as a tPA. Different from urokinase, the other endogenous plasminogen activator, tPA exhibits a notable affinity for fibrin and mediates efficient activation of plasminogen on the surface of blood clot.^6^,^8^ It is now clear that tPA is expressed in various tissues of the whole body. Under basal conditions, tPA is synthesised predominantly by vascular endothelial cells, then secreted into the blood, circulating as complexes with its physiological inhibitor PAI-1. In vivo, the concentration of tPA in plasma ranges from 5 to 10 ng/mL and exhibits significant variation under various physiological and pathological conditions.^7^

During the process of thrombolysis, the most important step is the degradation of high molecular weight fibrin into soluble low-molecular-weight products by fibrinolytic enzyme. Under normal physiological conditions, the content of plasmin in circulation is extremely low, but a large amount of plasmin can be formed locally after plasminogen is activated by plasminogen activators, of which tPA is the most important one in the fibrinolytic system. In normal circulation, tPA exhibits low activity towards plasminogen. However, this activity is enormously increased after tPA binds to the fibrin clot surface during thrombolysis, with efficient hydrolysis of the Arg561-Va1562 peptide bond of plasminogen on the clot surface, converting the zymogen into the active plasmin.^9^,^11^ Because of the value of t-PA for specific thrombolytic therapy, intensive efforts have been made to clarify the structural mechanisms and fibrinolytic properties responsible for its function.

#### Structure–function relationship in tPA

The newly synthesised protein of tPA contains 562 amino acids and is secreted into extracellular space in the form of a mature single-chain GP molecule after cleavage of the amino-terminal signal-peptide sequences. As shown in figure 1B, the tPA single-chain molecule consists of 527 amino acids (~70 kDa), with 17 disulfide bonds, one free sulfhydryl (Cys-83), 3 N-glycosylation (Asn117, Asn184, Asn448) and one *O*-glycosylation (Thr61). Five distinct modules are a finger domain (F, residues 4-50, homologous to the finger-like structures of fibronectin); an epidermal growth factor-like domain (E, residues 51-87, homologous to human and murine epidermal growth factor); two kringle domains (K1, residues 88-176 and K2, residues 177-256, homologous to the kringle structures of plasminogen) and a C-terminal trypsin-like proteolytic domain (P, residues 276-527), containing the active-site residues His 322, Asp371 and Ser 478.^8^ The F and the K2 modules play roles in the binding to fibrin. The F or the E modules and the carbohydrate side chains may be related to the rapid tPA clearance in vivo. The P domain is related to the enzymatic activity of tPA. In addition, Lys296-His-Arg-Arg299 is essential for the rapid inhibition of tPA by PAI-1. The 416th amino acid plays a pivotal role in maintaining the activity of single-chain tPA.^8^ In the presence of plasmin, the sensitive Arg275-Ile276 peptide bond in the native single-chain tPA is proteolytically cleaved, converting the single-chain into the two-chain form. Its single-chain form exhibits considerable catalytic activity to plasminogen, with an increase of catalytic efficiency only 5–10 fold after proteolytic cleavage to a two-chain form of tPA.^12^

#### Fibrinolytic properties of tPA

tPA has a highly specific affinity for fibrin. In the presence of tPA during the clotting of blood, plasma or purified fibrinogen, tPA binds almost completely to the clots. Under basic conditions, the activation of plasminogen by tPA is quite slow, with a Michaelis-Menten kinetics of K_m_=65 µmol/L and k_2_=0.06/s. However, when intravascular thrombosis occurs, the activation is enormously stimulated by fibrin and fibrin-related compounds, with a Michaelis-Menten kinetics of K_m_=0.16 µmol/L and k_2_=0.1/s.^6 9 13^ This effect forms the basis of the specific fibrinolytic properties of tPA. On the other hand, both tPA and plasminogen possess the ability to bind to fibrin. During the process of tPA-specific thrombolysis, the fibrin has a dual function, acting as a participant in plasminogen activation and as final substrate for plasmin formation. The tPA-induced lysis of a fibrin clot is characterised by two distinct phases. In the initial slow phase, single-chain tPA activates plasminogen on an intact fibrin surface. Subsequently, in the second rapid phase, fibrin undergoes partial degradation by plasmin, exposing additional binding sites for both plasminogen and tPA. This results in the single-chain tPA is cleaved to the more active two-chain form, with plasminogen continuously being converted to plasmin. Therefore, tPA, plasminogen and fibrin together create a cyclic ternary complex, amplifying the thrombolytic effect in the form of positive feedback cycle.^7^

It is important to note that the specificity of tPA is only observed at physiologic levels of tPA. However, at the pharmacological levels of tPA used in thrombolytic therapy, clot specificity is lost, leading to the establishment of a systemic lytic state and an associated increase in the risk of bleeding. In the coagulation cascade, endothelial cells synthesise and release PAI-1 to inhibit tPA; Additionally, α_2_ antiplasmin circulates in the blood at high concentrations, and under physiological conditions, it swiftly inactivates any plasmin that is not clot-bound. Nevertheless, due to the therapeutic doses of tPA, the regulatory system is overwhelmed, with bleeding as a direct consequence of the haemostatic fibrin lysis by tPA based on its specific fibrinolytic mechanism.

#### Physiological inhibitors of tPA

Being the most important PAI, PAI-1 serves as the primary physiological inhibitor of tPA. It efficiently inhibits both single-chain tPA and two-chain tPA as well as uPA. The circulating concentration of PAI-1 is approximately 20 ng/mL. The second-order rate constant for the inhibition of single-chain tPA by PAI-1 is 5.5×10^6^ M^−1^S^−1^, while the constant of two-chain tPA is 1.8×10^7^ M^−1^S^−1^, roughly three times higher than that of single-chain tPA.

PAI-2 was first demonstrated in human placental tissue extracts and was therefore named placenta-type PAI. Usually, the plasma concentration of PAI-2 is low, but it can rise to levels exceeding 35 ng/mL in pregnant women. The role of PAI-2 as tPA inhibitor is small, with slow inhibition of single-chain tPA (4.6×10^3^ M^−1^S^−1^) and a slight faster inhibition of two-chain tPA.^7^

In addition, other inhibitory proteins in plasma such as α_2_-antiplasmin, Cl-inactivator, α_1_-antitrypsin and α_2_-macroglobulin, show very slow inhibition of tPA, which may work after PAI-1 is exhausted.^7^

#### The role of tPA in neuronal survival

In addition to its essential role in fibrinolysis, tPA is also extensively expressed in the brain and is involved in the normal development and neuron function of brain. tPA-deficient mice display impaired memory, learning, visual processing along with reduced expression of smooth muscle cells in cerebral arteries, indicating a diminished response to neurovascular coupling regulation. When ischaemic stroke occurs, tPA is rapidly released in the ischaemic region, and there are still controversies about whether tPA plays a protective or deleterious roles in neuronal fate. Protective functions encompass antiexcitotoxicity, antiapoptosis and enhancement of energy supply. In contrast, its deleterious functions include the opening of blood–brain barrier, inflammation and excitotoxicity.^14^

#### Alteplase

In 1983, human tPA was first produced in *Escherichia coli*.^15^ Later, mammalian cell lines were used to produce human tPA. This recombinant human tPA (alteplase) was not different from natural human tPA in terms of biochemical properties and thrombolytic activity (table 1, figure 2). In 1990, alteplase was first used in patients with acute ischaemic stroke.^16^ After the publication of the seminal National Institutes of Neurological Disorders and Stroke trial in 1995,^17^ alteplase was approved by US Food and Drug Administration (FDA) for treating acute ischaemic stroke in 1996. Within 4.5 hours after onset of acute ischaemic stroke, intravenous thrombolysis (IVT) with alteplase is recommended or combined with endovascular thrombectomy (EVT), however, exceeding 4.5 hours only EVT is allowed.^18^ The recommended dosage of alteplase is 0.9 mg/kg (maximum 90 mg, 10% intravenous injection and 90% infusion over 60 min). The continuous infusion administration is due to rapid clearance of alteplase by the liver in the blood circulation, which results in a very short half-life of about 5 min.^9^ The absolute contraindication of alteplase includes prior intracranial haemorrhage, known structural cerebral vascular lesion, known malignant intracranial neoplasm, ischaemic stroke within 3 months, suspected aortic dissection, active bleeding or bleeding diathesis (excluding menses) and significant closed-head trauma or facial trauma within 3 months.

The major adverse effect of alteplase is haemorrhage, including symptomatic intracerebral haemorrhage and major systemic bleeding. This is attributed to the overwhelmed degradation of fibrin in haemostatic sites of vascular injury or the systemic lytic state resulting from the systemic generation of plasmin. In addition to haemorrhage, other adverse effects include allergy and angioedema, in which angioedema may be caused by using ACE inhibitors.^18^

Regrettably, despite being used in clinical use for more than 25 years, alteplase’s narrow therapeutic time window results in fewer than 5% of patients with acute ischaemic stroke receiving IVT globally in the eligible therapeutic time window.^19^ The thrombolysis rate only reaches about 20% in some advanced regions (average 5.64% in China).^20 21^ Several methods have been applied for increasing this ratio, such as offering remote assessment, increasing mobile stroke units and strengthening patient cognition of symptom onset, which could boost the diagnosis and treatment of acute ischaemic stroke.^4^

### Tenecteplase

Given the rapid clearance and increasing bleeding complication caused by alteplase, tenecteplase (a triple combination mutant variant) was generated in 1994. This variant, also known as TNK, involves substitutions: Thr103 to Asn (T mutation), Asn117 to Gln (N mutation) and the sequence Lys296-His-Arg-Arg to Ala-Ala-Ala-Ala (K mutation) (table 1, figure 2).^22^ These changes aim to reduce the clearance rate of the plasminogen activator (mutations T and N), enhance its resistance to PAI-1 and improve its fibrin specificity (mutation K), however, without improvement in fibrinolytic potency in vitro.^23^ In animal study, TNK sites mutagenesis showed 80-fold resistance to inhibition by PAI-1, a 14-fold increase in fibrin specificity and decreased plasma clearance rate.^22^

In 1999, a subset of patients with acute myocardial infarction received treatment with Tenecteplase, demonstrating early opening of infract-related coronary arteries compared with alteplase.^24^ Clinical trials proved the basic properties in human that tenecteplase has higher fibrin-specificity (10% reduction of fibrinogen and plasminogen vs 50% reduction in alteplase) and longer plasma half-life of approximately 22 min (alteplase about 5 min).^25^,^27^ It is interesting to note that the decreased rate of systemic clearance observed in animal and clinical trials enables the administration of tenecteplase as a rapid single bolus, offering an easier administration compared with alteplase. Subsequently, tenecteplase was approved by FDA and European Medical Agency in 2000 for weight-based treatment in ST-elevation myocardial infarction.

The first clinical study about tenecteplase in acute ischaemic stroke was published in 2005,^28^ and subsequent dosage studies and phase III clinical trials indicated that a dose of 0.25 mg/kg appeared to be safe and effective.^429^,^31^ Several randomised clinical trials comparing the safety and efficacy of tenecteplase with alteplase in patients with acute ischaemic stroke have suggested that tenecteplase is non-inferior to alteplase, even might precede in some aspects, such as ease administration, early recanalisation and smaller perfusion lesion volumes.^429^,^32^ Meanwhile, a meta-analysis including five randomised controlled trials showed higher rates of early neurological improvement in the tenecteplase group compared with alteplase group.^33^ These results support tenecteplase as a promising alternative thrombolytic agent even a replace for alteplase in treating acute ischaemic stroke.

### Staphylokinase and its recombinant non-immunogenic staphylokinase

Staphylokinase was first described to have fibrinolytic property in 1948^34^ and produced recombinantly in 1983 (table 1, figure 2).^35^ This protein does not function as an enzyme but indirectly activates plasminogen in a fibrin-selective way. In recent clinical trial of treating acute ischaemic stroke, recombinant non-immunogenic staphylokinase, a variant of staphylokinase, is non-inferiority to alteplase.^36^ Depending on its highly fibrin-selective and low cost (bacterial origin and small size), non-immunogenic staphylokinase holds promise to develop as a new thrombolytic agent.

The staphylokinase gene encoding 163 amino acids was cloned from certain strains of *Staphylococcus aureus*.^35 37^ The mature staphylokinase comprises 136 amino acids with a molecular weight of 16.5 kDa in a single polypeptide chain without disulfide bridges.^37^ Staphylokinase does not directly function as an enzyme to activate plasminogen but instead forms a 1:1 stoichiometric complex with plasmin(ogen), subsequently activating other plasminogen.^38 39^ In this model, plasminogen and staphylokinase (Plg.Sak) could transform into plasmin.staphylokinse (Pli.Sak) in a rate-limiting step. This transformation is accelerated by plasminogen activators (eg, Pli.Sak) and delayed by plasmin inhibitors (eg, α_2_-antiplasmin). The generated complex Pli.Sak can then convert plasminogen to plasmin, this reaction obeys Michaelis-Menten kinetics with K_m_=7.0 µmol/L and k_2_=1.5/s.^39^ The molecular structure study demonstrated that the residues 26, 42–50, 65–69 and 75 are critical for the Pli.Sak binding,^40^ while residues 11–16, 46–50, 65–69 and 97–98 are crucial for the processing and binding of the plasminogen.^40^ Its high fibrin-selective in human plasma milieu is attributed to the mutual action of fibrin and α_2_-antiplasmin, that is, in the absence of fibrin, the Pli.Sak would be inhibited by α_2_-antiplasmin, while in the presence of fibrin, the inhibition would be reduced more than 100-fold.^41^

In vivo animal study, staphylokinase acts as a thrombolytic agent without causing significant systemic depletion of fibrinogen.^42 43^ In two small pilot studies,^44 45^ 8 of 10 patients with coronary artery occlusion, treated with intravenous injection of 10 mg staphylokinase (1 mg bolus and infusion of 9 mg over 30 min) showed complete recanalisation with a half-life of 6.3 min.^44^ In a randomised trial of acute myocardial infarction, staphylokinase showed as effective as alteplase for early coronary artery recanalisation. Moreover, staphylokinase exhibit a lesser procoagulant effect than alteplase, as indicated by unaltered levels of fibrinogen, plasminogen and α_2_-antiplasmin in plasma.^46^ No allergic reactions or other side effects were observed in this trial. However, in this trial and subsequent double-bolus of staphylokinase trial, the induction of circulating neutralising antibodies against staphylokinase was observed, persisting at elevated levels for several months.^47^

Given the biggest disadvantage of staphylokinase on immunoreactivity, a variant of recombinant staphylokinase with Lys74, Glu75 and Arg77 substituted with Ala was produced in 1996. This variant maintained intact thrombolytic potency while a 200-time reduction in antibodies.^48^ This non-immunogenic staphylokinase was registered in Russia in 2012 as a thrombolytic drug for the treatment of patients with ST-segment elevation myocardial infarction^49^ and in 2020 for the treatment of patients with acute ischaemic stroke.^36^ In a randomised trial of treating patients with acute ischaemic stroke, non-immunogenic staphylokinase (10 mg single bolus) was no-inferior to alteplase. In the non-immunogenic staphylokinase group, 84 (50%) patients achieved a favourable outcome at day 90 compared with 68 (40%) patients in the alteplase group. There were no significant differences in mortality, symptomatic intracranial haemorrhage or serious adverse events between the two groups.^36^

## Stem cells for stroke therapy

Stem cells are undifferentiated cells and characterised by their ability to divide and differentiate into multiple cell lineages, as well as their capacity for long-term self-renewal, which allows them to maintain their undifferentiated state and continue to generate daughter cells with the potential for differentiation.^50^ Their unique properties make them a valuable tool for regenerative medicine and tissue engineering applications.

Stem cells can be derived from diverse sources, including embryos, adult tissues such as bone marrow (BM) and adipose tissue, and blood.^51^ Embryonic stem cells possess broader differentiation capabilities but entail ethical considerations in their collection. Adult stem cells, sourced from accessible tissues, are more ethically accepted. Stem cells are typically stored through cryopreservation methods, involving either conventional freezing or liquid nitrogen freezing, ensuring prolonged viability and functionality.^52^ In addition, specialised stem cell banks exist to collect, preserve and distribute various stem cell types for research, medical and therapeutic purposes, adhering to ethical and regulatory standards.^53^ The availability of stem cells is influenced by sourcing methods, ethical considerations and regulatory frameworks, with ongoing technological advancements continually refining the collection, storage and application processes for enhanced prospects in medical research and treatment.^53^

Enhanced endogenous neurogenesis and angiogenesis poststroke have been shown to significantly enhance neurofunctional recovery; our related work on NAMPT also had similarly substantiated this claim.^154^,^56^ Numerous preclinical trials have demonstrated that stem cell transplantation after stroke can improve neurogenesis, synaptogenesis and angiogenesis in the brain, leading to functional recovery.^57^,^59^ Its beneficial effects are mediated by multiple mechanisms, mainly including cell replacement and paracrine effects.^60^ Stem cells have the ability to differentiate into various cell types, such as neurons and glial cells, which can replace dead or damaged cells in the affected area, thereby restoring brain tissue function.^61^ Additionally, stem cells can secrete a variety of bioactive molecules, such as growth factors, cytokines and extracellular vesicles, which can promote angiogenesis, neurogenesis and synaptogenesis, and modulate immune and inflammatory responses, ultimately leading to tissue repair and regeneration.^62^ Some new mechanisms are also being uncovered, for example, a recent study found that mononuclear cells (MNCs) facilitated the uptake of vascular endothelial growth factor by endothelial cells and concurrently inhibited autophagy via gap junction-mediated intercellular interaction.^63^

### Clinical trials of stem cell therapy for stroke

Over the past decades, stem cell transplantation has been recognised as very promising therapeutic avenue with significant potential for the treatment of stroke including haemorrhagic stroke and ischaemic stroke. Numerous global clinical trials have investigated the therapeutic potential of different types of stem cell for the treatment of stroke (table 2, more detailed data in online supplemental material). In terms of reported clinical trials to date, the majority of clinical trials conducted are only phase I or II, and only a small proportion of trials have established a control group consisting of either non-blinded or blinded patients.^64^ What’s more, there is significant variation in the methods used across different trials. Regarding the outcomes of these clinical trials, all studies on stem cell therapy for stroke, except for one that used xenogeneic pig cells and reported adverse effects such as seizures and deterioration of motor function,^65^ did not report any harmful outcomes.

**Table 2:** Major clinical trials of cell-based therapies for stroke: summary of therapeutic time windows and outcomes

Cell type	Route	Stroke type and phase	Therapeutic time window (time post stroke)	Major outcome
Autologous BM-MNC	IV/IA	Acute ischaemic stroke	1–9 days	Safe
	IV/IA	Subacute ischaemic stroke	1–4 weeks	Safe, improvement of neurological function
	IC	Haemorrhagic stroke	5–7 days	Safe, improvement of neurological function
Autologous BM-MSC	IV	Subacute ischaemic stroke	1–2 months	Safe
Allogeneic BM-MSC	IV/IC	Chronic ischaemic stroke	6 months to 25 years	Safe, improvement of neurological function
Autologous BM-MNC/BM-MSC	IC/ICV	Haemorrhagic stroke	3–28 days	Safe, short-term improvement of neurological function
Allogeneic NSC (CTX0E03)	IC	Chronic ischaemic stroke	2–60 months	Safe, improvement of neurological function
Allogeneic multipotent adult progenitor cell	IV	Acute ischaemic stroke	1–2 days	Safe
Autologous BMSC and EPC	IV	Acute ischaemic stroke	1 month	Safe

The phase of strokes in table 2 is based on the actual staging descriptions of recruited patients who had a stroke in various clinical trials. Please refer to online supplemental table for details.

BM-MNC, bone marrow mononuclear cell; BM-MSC, BM mesenchymal stem cell; BMSC, BM stem cell; EPC, endothelial progenitor cell; IA, intraarterial; IC, intracerebral; ICVintracerebroventricularIV, intravenous; MSC, mesenchymal stem cell; NSC, neural stem cell

#### Cell types of administration

Several types of stem cell, such as MNCs, mesenchymal stromal cells (MSCs) and neural stem cells (NSCs), have been extensively studied and tested in clinical trials. It is important to note that certain clinical trials use not just a single cell type but may encompass a variety of cell types. According to the source of cells, they can be divided into autologous stem cells and allogeneic stem cells. Autologous cells present the benefit of minimal risk for post-transplant rejection and allergies, while allogeneic cells are advantageous due to their accessibility facilitated by large-scale production and the availability of standardised stocks.^66^

Autologous BM-MNCs are the most commonly used cell type in cell therapy for stroke, with the advantages of wide availability, easy accessibility and no immune rejection. Around 10^8^ MNCs can be acquired from 50 mL of BM and can be promptly transplanted for after isolation.^60^ It is worth noting that BM-MNCs represent a diverse collection of cells comprising differentially matured B-cells, T-cells and monocytes, alongside a smaller fraction of progenitor cells, including haematopoietic stem cells, MSCs and endothelial progenitor cells.^67^ Based on clinical trials, BM-MNCs have been demonstrated to be safe and feasible for intra-arterial, intravenous, intracerebral or intrathecal administration in patients who had a stroke.^68^ However, their effectiveness requires further confirmation through larger-scale clinical trials.

Autologous BM-MSCs are the second most commonly used cell type in stem cell therapy for stroke. BM-MSCs possess the capability for self-renewal, characterised by the expression of markers associated with mesenchymal cells and endothelial cells (CD105, CD73 and CD90), in addition to adhesion molecules (CD106, CD166 and CD29).^60^ BM-MSCs hold various advantages compared with other stem cells, attributed to their well-established harvesting techniques, minimal risk of tumourigenicity, distinct immune tolerance and lack of ethical concerns.^57^ Gene modifications of BM-MSCs also have been reported, including the development of SB623 cells by SanBio. This was accomplished by transitorily introducing a plasmid carrying the human Notch-1 intracellular domain, leading to amplified neuroprotective characteristics, including increased secretion of trophic factors, heightened anti-inflammatory impact and the stimulation of neurogenesis and angiogenesis.^69^ In the treatment of chronic cerebral ischaemic diseases (NCT01287936), SB623 cells had been demonstrated reliable safety and favourable clinical outcomes. However, the primary outcome of clinical study (NCT02448641) on SB623 in patients with chronic motor deficits from ischaemic stroke did not show significant effectiveness of the treatment.

NSCs possess the remarkable ability to differentiate into neural cells, oligodendrocytes and astrocytes, allowing for seamless integration into the host brain. This inherent capability makes NSCs a promising therapy for stroke.^70^ Despite the potential of NSCs to serve as a suitable option for replenishing lost neuronal networks, their procurement from fetal sources presents ethical concerns. CTX0E03 is a human NSC line derived from embryonic brain tissue. It has been subjected to immortalisation through retroviral transduction of the c-myc growth factor gene, resulting in sustained self-renewal and maintenance of stem cell properties.^71^ CTX-DP is a commercial product derived from the CTX0E03 cell line and has been used for the treatment of chronic stroke. The results of the PISCES I trial (NCT01151124) provided evidence of the safety and tolerability of CTX0E03.^72^ The PISCES 2 study (NCT02117635) enrolled adults over 40 years of age with significant upper limb motor deficits 2–13 months (median: 7 months) post-ischaemic stroke.^73^ Overall, no safety concerns were reported and 15 patients exhibited improvement on one or more clinical assessment scales.^73^ The PISCES trial was further progressed into PISCES III. However, due to the prevailing COVID-19 pandemic and its associated epidemiological situation, the project experienced a temporary suspension.

Alternative cell sources, including umbilical cord blood cells, adipose-derived stem/stromal cells and neuronal progenitor cells, are also used in clinical applications. Several fundamental studies have conducted comparisons regarding the efficacy of diverse cell sources as therapeutic approaches.^69 74^ Nevertheless, each type of stem cell possesses its inherent advantages and limitations, and presently, it remains uncertain which cell type embodies the most advantageous treatment.

#### Routes and dosage of administration

The transplantation procedure entails the direct injection of stem cells into the affected brain tissue using intracerebroventricular and intracerebral routes, or systemic infusion via intravenous and intra-arterial administration. In terms of invasiveness, intravenous transplantation offers a significant advantage as it is minimally invasive, enabling the possibility of multiple injections. However, the intravenous transplantation of stem cells for stroke treatment often leads to a restricted number of cells reaching the damaged lesion, as a significant portion of the transplanted cells become trapped in the lungs.^75^ In addition, intravenous route faces challenges such as microthrombus formation, lack of targeting specificity and potential immune rejection.^76^ The intra-arterial approach is regarded as superior to intravenous administration in terms of delivering a higher number of cells to the lesion site, but it may potentially lead to complications such as vascular obstruction and embolism, further compromising cerebral blood supply.^77^ The risk is often linked to MSCs due to their relatively larger size, but the actual incidence rate in clinical practice is infrequent.^78 79^ The method of intracerebral administration, involving direct cell delivery, results in the highest degree of cell engraftment. However, it necessitates invasive surgery and poses the risk of potential further brain injury. Compared with the intracerebral approach, intrathecal injections are less invasive, but the rate of cell engraftment remains unclear, and there is a risk of obstructive hydrocephalus. In the acute to subacute phase, intravenous transplantation appears to be the preferred method, whereas intracerebral or intracerebroventricular transplantation is predominantly performed during the chronic phase.

Other delivery methods being explored for stroke treatment include intranasal delivery, which involves delivering stem cells through the nasal cavity to the brain and hydrogel-based delivery, which involves embedding stem cells in a biocompatible scaffold that can be injected into the brain tissue.^80^ These methods have the potential to improve stem cell delivery and distribution in the brain while minimising invasiveness and risks.

The dosages of stem cell transplantation for stroke treatment typically range from 10^6^ to 10^9^ cells and can vary based on multiple factors, including the stem cell type used, the administration route, the timing of transplantation, and the specific clinical trial or the treatment protocol being implemented. In the acute and subacute phases of stroke, intravascular routes provide a considerable cell count of up to 10^9^ cells, whereas intracerebral routes offer a smaller cell dosage ranging from 10^6^ to 10^7^ cells in the chronic phase. However, it is noteworthy that there is currently no consensus regarding the therapeutic cell dosage in stem cell transplantation for the treatment of stroke. For detailed information on the actual cell dosages and administration routes employed in cell therapy for stroke, please refer to online supplemental table.

#### Timing of administration and outcome measure

In clinical practice, stroke can be classified into three stages based on the therapeutic time window and degree of pathological changes: acute, subacute and chronic stages. The acute stage typically refers to the period from a few days to 1 month after onset; the subacute stage usually spans from a few weeks to 6 months after onset; and the chronic stage generally encompasses the period of 6 months or longer after onset.^81^ The timing of stem cell transplantation after stroke is critical for maximising therapeutic outcomes. The therapeutic time window for stem cell transplantation in the treatment of stroke refers to a specific period of time following the onset of stroke during which the transplantation is performed to achieve optimal therapeutic effects. Current clinical research suggests that the therapeutic time window for stem cell transplantation in stroke may vary depending on the specific stem cell type and treatment approach.^82^ Generally, early transplantation within a few hours to several days after stroke onset is considered the optimal therapeutic time window.^83^ However, some studies have also indicated that extending the therapeutic time window to several weeks or even months may still yield some therapeutic benefits.^84^ Nevertheless, the exact therapeutic time window is still under further exploration and determination in clinical research. Therefore, in practical treatment, the determination of the therapeutic time window for stem cell transplantation in stroke should be based on individual assessment and decision-making.

As for the outcome measure, there was a significant variation in the assessment modalities used across trials. However, the modified Rankin Scale, National Institute of Health Stroke Scale and Barthel index emerged as frequently employed measures.

### The challenge of stem cell therapy for stroke

**Figure 3:**
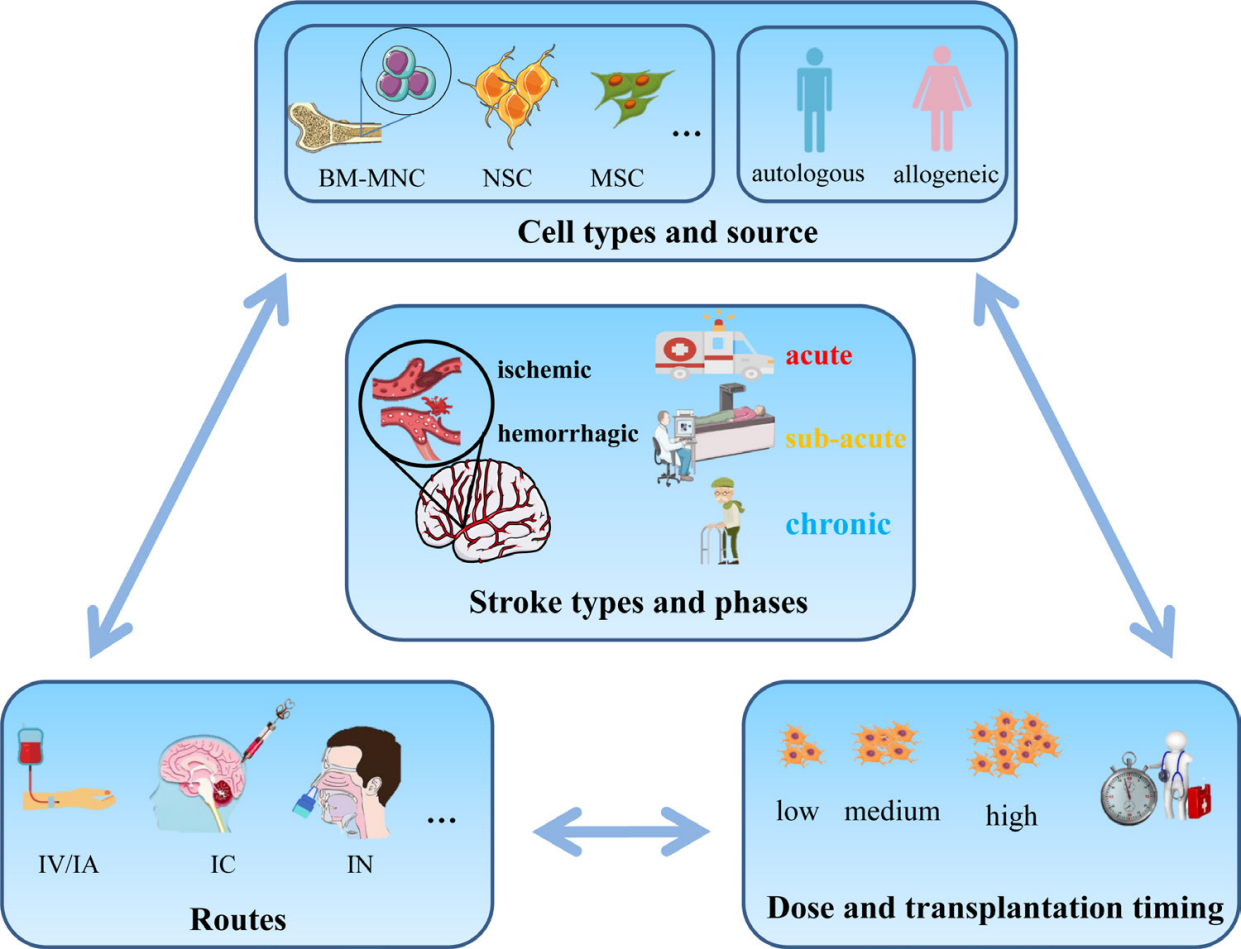
Challenges in stem cell transplantation for stroke therapy. The major challenge in stem cell transplantation for stroke treatment is to precisely select appropriate cell types, dose, transplantation timing and delivery routes based on the patient’s stroke type and stage, with the goal of ensuring safety while maximising the improvement of neurological function. BM-MNC, bone marrow mononuclear cell; IA, intraarterial; IC: intracerebral; IN, intranasal; IV, intravenous; MSC, mesenchymal stem cell; NSC, neural stem cell.

Although stem cell therapy for stroke displays promise as a potential treatment method, it presents various challenges that must be tackled for its successful application in clinical settings (figure 3). One major challenge is the identification of the optimal cell type and timing tailored to different stroke subtypes.^60 85^ Each cell type possesses distinct properties, including differentiation potential, immunogenicity and tumourigenicity. It is crucial to find the ideal cell type that balances safety, efficacy and practicality for different stroke subtypes. Simultaneously, determining the optimal timing for stem cell transplantation is vital. The therapeutic time window after stroke is limited, necessitating prompt intervention to maximise recovery chances. The timing for stem cell transplantation must consider various factors such as the inflammatory response, tissue damage and the potential for neuroplasticity. Identifying the ideal therapeutic time window of opportunity for transplantation remains an ongoing area of investigation.^60^

Another critical challenge is ensuring the survival, integration and appropriate differentiation of transplanted stem cells within the injured brain tissue.^86 87^ The harsh microenvironment, limited blood supply and the potential for immunological rejection pose obstacles to successful engraftment. Researchers are actively investigating strategies to enhance the survival of transplanted stem cells, facilitate their integration with existing neural networks and guide their differentiation into desired neural cell lineages.^64^ These approaches encompass gene modifications and preconditioning techniques, scaffold-based methodologies, combination therapies, immunomodulation and optimisation of timing and delivery methods.^60^

In addition, standardising protocols for stem cell preparation, quality control and delivery methods are also crucial for the widespread implementation of stem cell transplantation. Ensuring scalability and reproducibility of results across different clinical settings is essential to establish consistent and reliable outcomes. Furthermore, comprehensive evaluation and mitigation of safety risks, such as tumour formation, immune reactions and ectopic tissue formation, are paramount to ensure the safety of patients undergoing stem cell transplantation.

Addressing these challenges requires continued research, technological advancements and the establishment of robust regulatory frameworks. It is essential for scientists, clinicians, ethicists and regulatory authorities to collaborate in order to surmount these obstacles and facilitate the successful transition of stem cell transplantation into a safe and efficient therapy for individuals affected by stroke.

## Organoids for stroke therapy

Tissue transplantation, in comparison to cell transplantation, provides advantages such as preservation of structural integrity, diverse cellular composition, maintenance of the native microenvironment, potential for vascularisation, enhanced functional recovery and greater surgical feasibility, making it a more comprehensive and effective approach for tissue repair and regeneration. Cerebral organoids, also known as brain organoids or mini-brains, are three-dimensional clusters of brain-like tissue that are generated from human pluripotent stem cells in the laboratory.^88^ Cerebral organoids possess extensive cell numbers, diverse cell types and specific tissue architecture that can mimic some of the developmental processes and cellular interactions that occur in the developing brain, making them a valuable tool for studying brain development, disease modelling and potential therapeutic applications, including for stroke therapy.^88^,^92^

### Cerebral organoids transplantation

Improving the survival and integration of transplanted stem cells in injured brain tissue is crucial for the success of stem cell transplantation therapy in treating stroke, but it remains a major challenge. Limited cell survival, as well as poor integration and differentiation of transplanted cells with the host tissue, significantly impede the therapeutic potential of stem cell transplantation in stroke treatment. Unlike stem cell transplantation, cerebral organoids transplanted into the brain of immune-deficient mice can form blood connections with the host and exhibit robust blood flow.^93^ Another study also found that cerebral organoid grafts had higher rates of survival and more advanced vascularisation compared with transplants using dissociated neural progenitor cells.^94^ The pronounced vascularisation of cerebral organoids significantly promotes their survival and facilitates further growth and differentiation.

The transplanted cerebral organoids not only establish vascular connections with the host but also exhibit anatomical and functional integration with the host’s nervous system. Notably, engrafted cerebral organoids demonstrated spontaneous rhythmic calcium transients, neuronal action potentials and synchronised neuronal network activity, highlighting their remarkable capacity for functional integration.^93^ In addition, the organoid grafts exhibited robust axonal growth, established synaptic connections with host neurons and demonstrated long-distance axonal projections from the grafts to the host brain, indicating their extensive neural network integration capabilities.^93^

### Cerebral organoids for stroke and other brain injury therapy

Cerebral organoids, which possess considerable cell numbers, diverse neural cell types, organisational morphological characteristics, and enhanced survival and integration capabilities, offer potential advantages over stem cells for transplantation therapy. Therefore, subsequent research has increasingly emphasised the therapeutic potential of organoids transplantation. Our study conducted the first investigation into the therapeutic value of transplanting cerebral organoids for the treatment of traumatic brain injury and stroke. Our study discovered that transplanting cerebral organoids into the damaged motor cortex of rat supported region-specific reconstruction of damaged motor cortex, reduced brain damage and improved neurological motor function.^91^ Moreover, in the rat stroke model, our study demonstrated that cerebral organoids transplantation significantly reduced brain infarct volume and improved neurological motor function.^73^ We discovered that transplanted cerebral organoids underwent further differentiation, formed synaptic connections with the host brain and aided in the reconstruction of the damaged motor cortex in a region-specific manner.^73^ Transplantation therapy using cerebral organoids is linked to increased neurogenesis, synaptic reconstruction, axonal regeneration and angiogenesis. It is also associated with reduced neural apoptosis and higher neuronal survival following a stroke.^73^

Subsequent researches have delved more deeply into the functional integration between transplanted cerebral organoids and the host brain. One study discovered that cortical organoids derived from human stem cells, when transplanted into the somatosensory cortex of athymic rats, successfully integrated into circuits related to sensory perception and motivation. The transplanted organoids received inputs from thalamocortical and corticocortical pathways, and these inputs triggered sensory responses in the human cells.^95^ Two recent studies also highlight the remarkable findings that transplantation of human forebrain cortical organoids into the damaged visual cortex of adult rats not only established structural connectivity but also achieved sophisticated functional integration.^96 97^ These studies provide compelling evidence that human cerebral organoids have the capacity to integrate and form functional circuits within the host brain, suggesting the exciting therapeutic potential of cerebral organoids in restoring brain function following injury.

Though organoids encompass a broader range of cell types, which grants them certain advantages as disease models and transplantation donors, the presence of multiple cell types also introduces heightened uncertainty. Organoids transplantation encounters challenges such as tumourigenicity and immune rejection, necessitating further research to validate its safety. Moreover, organoids exhibit significant heterogeneity, wherein the cellular composition varies among individual organoids. The achievement of standardised production for organoids remains a significant challenge. Current research on organoids primarily takes place in laboratory settings, and there is still a long way to go before achieving organoids transplantation therapy for stroke.

## Conclusions and future directions

IVT is one approach for treating acute ischaemic stroke, and alteplase as a biological drug remains the only licensed thrombolytic agent. Current clinical trials show that two recombinant proteins, tenecteplase and non-immunogenic staphylokinase, are most promising as new thrombolytic agents for acute ischaemic stroke therapy. Another promising thrombolytic agent for treatment of acute ischaemic stroke is the recombinant human prourokinase that showed non-inferior to alteplase in China (phase 3, NCT03541668).^98^ The ideal thrombolytic agent should have several advantages including rapid reperfusion, low risk of haemorrhage, long half-life to allow single bolus administration, prevention of reocclusion and low cost. For this purpose, it is valued for future research to explore totally novel thrombolytic agent with novel mechanisms different from plasminogen activation, for example, directly cleaving fibrin without plasminogen activation.^40^

Stem cell-based therapy, employing stem cells or organoids for stroke treatment, has demonstrated promising results in preclinical and early clinical studies. Additional research is necessary to refine cell delivery techniques, identify the optimal cell type and dosage, and tackle long-term safety considerations. Future directions in stem cell-based therapy for stroke should focus on conducting large-scale clinical trials to establish the efficacy and safety of different stem cell approaches.

Additionally, due to the advent of highly successful reperfusion, there is an opportunity to explore combination therapies involving neuroprotective agents, rehabilitation strategies and genetic engineering techniques, which lead to improved stroke outcomes.^99^

## supplementary material

10.1136/svn-2023-002883 online supplemental file 1
