# Supplementary material for: Anti-stroke biologics: from recombinant proteins to stem cells and organoids
Source: Stroke Vasc Neurol. 2024 Jan 29;9(5):e002883. doi: 10.1136/svn-2023-002883 (PMC11732845; doi:10.1136/svn-2023-002883)
Supplement: online supplemental file 1 [file svn-9-5-s001.pdf]

Supplementary Information

Supplementary Table. Major clinical trial of stem cells for stroke therapy.

Cell type	Dose (cell number)	Route	Transplant timing after stroke	Treated patient number (Control)	Experimental design (NCT number)	Follow-up time	Functional outcome measure	Major outcome
**Acute ischemic stroke**								
Allogeneic multipotent adult progenitor cell	0.4×10^9; 1.2×10^9	IV	1-2 days	65 (61)	Randomized double-blind phase II trial (NCT01436487)	12 month	mRS, NIHSS, BI	Safe; but no significant improvement of neurological function[1]
Autologous BM-MNC	$7\text{-}10\times10^6$/kg	IV	1-3 days	10	Open-label prospective trial	6 month	mRS, NIHSS, BI	Safe[2]
Autologous BM-MNC	1.6×10^8	IA	5-9 days	10 (10)	Assessor-blind phase I/II trial	6 month	mRS, NIHSS, BI	Safe[3]
Autologous BM-MNC	2×10^6 /kg; 5×10^6 /kg	IA	1-7 days	38(38)	Randomized assessor-blind phase II trial (NCT02178657)	2 years	mRS, NIHSS	Safe; but no significant improvement in neurological function[4]

Autologous BMSC and EPC	3×10^8	IV	1 month	12 (6)	Randomized assessor-blind phase I/IIa trial (NCT01468064)	4 years	mRS, NIHSS, BI	Safe, but no significant improvement of neurological function[5]
**Sub-acute ischemic stroke**								
Autologous BM-MNC	2.8×10^8	IV	18 days	60(60)	Randomized assessor-blind phase II trial (NCT0150177)	12 months	mRS, NIHSS, BI	Safe, but no significant improvement of neurological function[6]
Autologous BM-MNC	1×10^6	IA	2-4weeks	21(18)	Open-label randomized assessor-blind trial	12 months	mRS, NIHSS, BI	Safe, but no significant improvement of neurological function[7]
Autologous BM-MNC	5×10^8	IA	1-2 weeks	10 (10)	Open-label prospective randomized assessor-blind trial	6 months	mRS, NIHSS, BI	Safe, improvement of neurological function[8]
Autologous BM-MNC (ALD-401)	3×10^6	IA	2-3weeks	29 (17)	Randomized assessor-blinded phase II trial (NCT01273337)	12 months	mRS, NIHSS, BI	Safe, but no significant improvement of neurological function[9]
Autologous BM-MSC	2×10^6 /kg	IV	1-2 months	9 (8)	Randomized assessor-blinded phase II trial (NCT01461720)	12 months	mRS, NIHSS, BI	Safe; but no significant improvement of neurological function[10]
Autologo	1×10^8;	IV	1-2	16(15)	Open-label	2 years	mRS,	Improvement of motor

us BM-MSC	3×10^8		months		randomized phase II trial (NCT 00875654)		NIHSS, BI	function[11]
Autologous MSC	1×10^8	IV	2 months	16(36)	Open-label randomized, assessor-blind trial	5 years	mRS, Survival rate	Safe, improvement of neurological function, less mortality for 5 years[12]
**Chronic ischemic stroke**								
NSC (CTX0E03)	2×10^6; 5×10^6; 10×10^6; 20×10^6	IC	6-60 months	12	Open-label phase I trial (NCT01151124)	2 years	NIHSS, BI, Ashworth	Safe, improvement of neurological function[13]
NSC (CTX0E03)	20×10^6	IC	2-12 months	21	Open-label prospective phase II trial NCT02117635	12 months	ARAT	Safe, improvement of upper limb function[14]
Allogeneic BM-MSC (SB623 cell)	2.5×10^6; 5.0×10^6; 10×10^6	IC	6-60 months	18	Open-label phase I/IIa trial (NCT01287936)	2 years	ESS, NIHSS, FM	Safe, improvement of neurological function[15]
Allogeneic BM-MSC (SB623 cell)	2.5×10^6 ; 5.0×10^6	IC	6-90 months	104(52)	Randomized double-blind phase II trial (NCT02448641)	12 months	FMMS, mRS, ARAT	No significant improvement in neurological function
Allogenei	0.5×10^6/kg;	IV	7	36	Randomized phase	12 months	NIHSS,	Safe, improvement of

c BM-MSC (hypoxia treated)	1×10^6/kg; 1.5×10^6/kg		months-2 5 years		I/II trial (NCT01297413)		BI	neurological function[16]
**Hemorrhagic stroke**								
Autologous BM-MNC	0.25-2.3×10^7	IC	5-7day	60(40)	Assessor-blind phase I trial	6 months	NIHSS, BI	Safe, improvement of neurological function[17]
Autologous BM-MNC / BM-MSC	MNC:0.17-1.5×10^6; MSC:1.7-3.2×10^4	IC (MNC); IT (MSC)	3-28 day	100(96)	Assessor-blind phase I trial	12 months	NIHSS, RS, BI	Safe, short-term improvement of neurological function[18]

BM-MNC: Bone marrow mononuclear cell; MSC: Mesenchymal stem cell; BM-MSC: Bone marrow mesenchymal stem cell; BMSC: Bone marrow stem cell; EPC: Endothelial progenitor cell; NSC: Neural stem cell; IV: intravenous; IA: intraarterial; IC: intracerebral; IT: intracerebroventricular; mRS: modified Rankin Scale, NIHSS: National Institute of Health Stroke Scale; BI: Barthel Index; ESS: European Stroke Scale; ARAT: Action Research Arm Test; FM: Fugl-Meyer Assessment; FMMS: Fugl-Meyer Motor Total Score; RS: Rankin scale. Note: The staging of strokes in Supplementary Table is based on the actual staging descriptions of recruited stroke patients in various clinical trials.

Reference

1 Hess DC, Wechsler LR, Clark WM, *et al.* Safety and efficacy of multipotent adult progenitor cells in acute ischaemic stroke (MASTERS): a randomised, double-blind, placebo-controlled, phase 2 trial. *Lancet Neurol* 2017;16:360-68.

2 Savitz SI, Misra V, Kasam M, *et al.* Intravenous autologous bone marrow mononuclear cells for ischemic stroke. *Ann Neurol* 2011;70:59-69.

3 Moniche F, Gonzalez A, Gonzalez-Marcos JR*, et al.* Intra-arterial bone marrow mononuclear cells in ischemic stroke: a pilot clinical trial. *Stroke* 2012;43:2242-4.

4 Moniche F, Cabezas-Rodriguez JA, Valverde R*, et al.* Safety and efficacy of intra-arterial bone marrow mononuclear cell transplantation in patients with acute ischaemic stroke in Spain (IBIS trial): a phase 2, randomised, open-label, standard-of-care controlled, multicentre trial. *Lancet Neurol* 2023;22:137-46.

5 Fang J, Guo Y, Tan S*, et al.* Autologous Endothelial Progenitor Cells Transplantation for Acute Ischemic Stroke: A 4-Year Follow-Up Study. *Stem Cells Transl Med* 2019;8:14-21.

6 Prasad K, Sharma A, Garg A*, et al.* Intravenous autologous bone marrow mononuclear stem cell therapy for ischemic stroke: a multicentric, randomized trial. *Stroke* 2014;45:3618-24.

7 Ghali AA, Yousef MK, Ragab OA*, et al.* Intra-arterial Infusion of Autologous Bone Marrow Mononuclear Stem Cells in Subacute Ischemic Stroke Patients. *Front Neurol* 2016;7:228.

8 Bhatia V, Gupta V, Khurana D*, et al.* Randomized Assessment of the Safety and Efficacy of Intra-Arterial Infusion of Autologous Stem Cells in Subacute Ischemic Stroke. *AJNR Am J Neuroradiol* 2018;39:899-904.

9 Savitz SI, Yavagal D, Rappard G*, et al.* A Phase 2 Randomized, Sham-Controlled Trial of Internal Carotid Artery Infusion of Autologous Bone Marrow-Derived ALD-401 Cells in Patients With Recent Stable Ischemic Stroke (RECOVER-Stroke). *Circulation* 2019;139:192-205.

10 Law ZK, Tan HJ, Chin SP*, et al.* The effects of intravenous infusion of autologous mesenchymal stromal cells in patients with subacute middle cerebral artery infarct: a phase 2 randomized controlled trial on safety, tolerability and efficacy. *Cytotherapy* 2021;23:833-40.

11 Jaillard A, Hommel M, Moisan A*, et al.* Autologous Mesenchymal Stem Cells Improve Motor Recovery in Subacute Ischemic Stroke: a Randomized Clinical Trial. *Transl Stroke Res* 2020;11:910-23.

12 Lee JS, Hong JM, Moon GJ*, et al.* A long-term follow-up study of intravenous autologous mesenchymal stem cell transplantation in patients with ischemic stroke. *Stem Cells* 2010;28:1099-106.

13 Kalladka D, Sinden J, Pollock K*, et al.* Human neural stem cells in patients with chronic ischaemic stroke (PISCES): a phase 1, first-in-man study. *Lancet* 2016;388:787-96.

14 Muir KW, Bulters D, Willmot M*, et al.* Intracerebral implantation of human neural stem cells and motor recovery after stroke: multicentre prospective single-arm study (PISCES-2). *J Neurol Neurosurg Psychiatry* 2020;91:396-401.

15 Steinberg GK, Kondziolka D, Wechsler LR*, et al.* Two-year safety and clinical outcomes in chronic ischemic stroke patients after implantation of modified bone marrow-derived mesenchymal stem cells (SB623): a phase 1/2a study. *J Neurosurg* 2018:1-11.

16 Levy ML, Crawford JR, Dib N*, et al.* Phase I/II Study of Safety and Preliminary Efficacy of Intravenous Allogeneic Mesenchymal Stem Cells in Chronic Stroke. *Stroke*

2019;50:2835-41.

17 Li ZM, Zhang ZT, Guo CJ*, et al.* Autologous bone marrow mononuclear cell implantation for intracerebral hemorrhage-a prospective clinical observation. *Clin Neurol Neurosurg* 2013;115:72-6.

18 Zhu J, Xiao Y, Li Z*, et al.* Efficacy of Surgery Combined with Autologous Bone Marrow Stromal Cell Transplantation for Treatment of Intracerebral Hemorrhage. *Stem Cells Int* 2015;2015:318269.
